# The Value of Ketone Bodies in the Evaluation of Kidney Function in Patients with Type 2 Diabetes Mellitus

**DOI:** 10.1155/2021/5596125

**Published:** 2021-04-10

**Authors:** Yimei Li, Yongze Zhang, Ximei Shen, Fengying Zhao, Sunjie Yan

**Affiliations:** ^1^Department of Endocrinology, The First Affiliated Hospital of Fujian Medical University, 20 Cha Zhong Road, Fuzhou, Fujian 350005, China; ^2^Diabetes Research Institute of Fujian Province, 20 Cha Zhong Road, Fuzhou, Fujian 350005, China; ^3^Metabolic Diseases Research Institute, The First Affiliated Hospital of Fujian Medical University, 20 Cha Zhong Road, Fuzhou, Fujian 350005, China; ^4^Fujian Province Clinical Research Center for Metabolic Diseases, 20 Cha Zhong Road, Fuzhou, Fujian 350005, China

## Abstract

**Objectives:**

Recent studies have shown that the slightly elevated circulating levels of ketone bodies (KBs) played a significant role in the treatment of various diseases. This study is aimed at investigating the association between different levels of KBs and kidney function in patients with type 2 diabetes mellitus (T2DM).

**Methods:**

A retrospective study of 955 patients with T2DM (426 women and 529 men) admitted to our hospital from December 2017 to September 2019 was conducted. Patients were divided into different groups in line with the levels of KBs (low-normal group: 0.02-0.04 mmol/L, middle-normal group: 0.05-0.08 mmol/L, high-normal group: 0.09-0.27 mmol/L, and slightly elevated group: >0.27 and <3.0 mmol/L).

**Results:**

In the present study, individuals with high-normal levels of KBs had the lowest risk of diabetic kidney disease (DKD) and increased peak systolic velocity (PSV); those with middle-normal levels of KBs had the lowest risk of increased renal arterial resistive index (RI), with a positive correlation between increased *α*1-microglobulin and KB concentration. In addition, the indicators of glomerulus, renal tubules, and renal arteries were all poor with slightly elevated circulating levels of KBs, and KB concentration lower than 0.09 mmol/L can be applied as the threshold for low risk of renal function damage.

**Conclusions:**

In summary, slightly elevated circulating levels of ketone bodies are not of benefit for renal function in patients with type 2 diabetes mellitus.

## 1. Introduction

Diabetic kidney disease (DKD) is one of the most important complications of diabetes, as well as the main reason of end-stage renal disease and renal replacement therapy, which remarkably reduces the quality of life and increases the mortality of diabetic patients [1]. DKD risk factors can be classified as irreversible factors (e.g., age, sex, and duration of diabetes) and modifiable factors (e.g., hyperglycemia, hypertension, dyslipidemia, and smoking) [2]. Besides, the established protective factors of DKD include blood sugar control, blood pressure control, and reduction of urine protein.

Ketone bodies (KBs) are produced predominantly in the liver and are then transported to extrahepatic tissues [3, 4]. Interestingly, renal ketogenesis has been inferred in diabetic animal models and is regarded as a protective mechanism to prevent renal ischemia-reperfusion injury [5]. Sodium-glucose cotransporter 2 (SGLT-2) inhibitors are a novel class of oral hypoglycemic drugs used for the management of type 2 diabetes mellitus (T2DM). A relevant feature of SGLT-2 inhibitors in the treatment of diabetic patients is an increase in circulating levels of KBs, which plays a significant role in the cardiovascular and kidney protection because of the use of KBs as energy substrates and their potential interactions with G-protein coupled receptors and other signaling pathways [6]. In addition, recent clinical studies have demonstrated that slightly elevated circulating levels of KBs play a significant role in the treatment of various diseases, such as heart failure, neurodegenerative disease, and cancer [3, 4, 6, 7]. However, some studies suggested that microvascular complications were found to be increased in diabetic patients who had elevated ketones compared to those with normal ketone levels [8].

To date, there are limited number of studies that have concentrated on the potential relationship between KBs and kidney in T2DM patients. To our knowledge, *β*-hydroxybutyric acid (*β*HBA) accounted for 78% of the KB component [9]; therefore, the concentration of *β*HBA is referred for the concentration of KBs. The present study is aimed at investigating the relationship between different levels of KBs and kidney in patients with T2DM and evaluating whether the slightly elevated circulating levels of KBs have a positive influence on kidney in patients with T2DM.

## 2. Materials and Methods

### 2.1. Study Population

In this study, a total of 955 patients (426 women and 529 men) were recruited from the Endocrinology Department of the First Affiliated Hospital of Fujian Medical University from December 2017 to September 2019 who met the 1999 World Health Organization (WHO) diagnostic criteria for type 2 diabetes and have the data of ketone bodies. (Supplemental Fig. 1). The diagnosis of T2DM was diagnosed based on the criteria presented by the American Diabetes Association [10]. Exclusion criteria were type 1 diabetes, gestational diabetes, diabetic ketoacidosis [11], hyperosmolar nonketotic coma, hepatic failure, fasting [12], patients who received SGLT-2 inhibitors, or who lacked data related to the levels of KBs. This study was approved by the ethics committee of the First Affiliated Hospital of Fujian Medical University, and all of the participants provided signed informed consent.

### 2.2. Measurement

Data describing the demographic characteristics, the duration of disease onset, lifestyle and medical histories, and medication histories were collected from medical records. Blood pressure (BP) was measured after 15 min of rest. Body weight and height were measured with the patient barefoot and wearing light clothes. Body mass index (BMI, kg/m^2^) was defined as weight in kilograms divided by square of the height in meters. The following factors were measured in all study subjects after 10 h of overnight fasting: fasting plasma glucose (FPG), triglycerides (TG), total cholesterol (TC), low-density lipoprotein cholesterol (LDL-C), serum creatinine concentration (SCr), and *β*HBA, as well as routine hematological and biochemical parameters. Glycated hemoglobin A1c (HbA1c) level was detected by high-performance liquid chromatography (HPLC; VARIANTTM II; BIO-RAD Laboratories Inc., Hercules, CA, USA). A 75 g oral glucose tolerance test (OGTT) was performed as well. Plasma glucose was measured by glucose oxidase method using the AU2700 analyzer (Olympus Corporation, Tokyo, Japan) and specific reagents. The urinary albumin-to-creatinine ratio (UACR) was calculated as albumin concentration (mg/L) divided by creatinine concentration (g/L) [10]. The eGFR was calculated by the CKD-EPI creatinine equation (2009) that can be expressed as a single equation: 141 × min (SCr/*κ*, 1)^*α*^ × max(SCr/*κ*, 1)^−1.209^ × 0.993^Age^ [×1.018 if female] [×1.159 if black], where SCr is serum creatinine, *κ* is 0.7 for females and 0.9 for males, *α* is −0.329 for females and−0.411 for males, min is the minimum of SCr/*κ* or 1, and max is the maximum of SCr/*κ* or 1 [13]. Urine protein (PRO) and urine specific proteins, including *α*1-microglobulin (*α*1-MG), urinary immunoglobulin G (U-IgG), and urinary transferrin (TRU), were measured from the first-void of the early morning urine sample. Renal vascular parameters were determined by color Doppler ultrasound (DEXA, Lunar Prodigy scanner; GE Lunar Corporation, Madison, WI, USA).

### 2.3. Diagnostic Criteria

DKD was diagnosed by the presence of eGFR < 60 mL/min and/or UACR > 30 mg/g for ≥3 months. [10, 12–14]. Hypertension (HT) was defined as systolic BP (SBP) ≥ 140 mmHg and/or diastolic BP (DBP) ≥ 90 mmHg and/or treatment with antihypertensive drugs [10, 15, 16]. Reduced renal artery diameter was identified as arterial diameter of <0.5 cm [17]. Increased peak systolic velocity (PSV) was defined as peak renal arterial blood flow rate of >100 cm/s [18, 19]. Increased renal arterial resistive index (RI) was calculated as RI > 0.7 [20]. In the present study, reference interval for *α*1-MG is lower than 15 mg/L [21]. Furthermore, we defined *α*1 − MG > 15 mg/L as increased *α*1-microglobulin. Renal function damage was defined as damage to one of the glomerulus, renal tubules, and renal arteries.

### 2.4. Statistical Analysis

The SPSS 20.0 software (IBM, Armonk, NY, USA) was used to perform statistical analysis. Continuous variables were expressed as mean ± standard deviation or median (P25–P75), and categorical variables were presented as count and percentage. The patients within the normal range of KBs were divided into tertiles (low-normal KB group: 0.02-0.04 mmol/L; middle-normal KB group: 0.05–0.08 mmol/L; high-normal KB group: 0.09–0.27 mmol/L). In addition, the slightly elevated KB concentration was higher than the normal concentration and lower than the concentration of ketoacidosis. Therefore, the slightly elevated circulating levels of KBs were defined as greater than 0.27 mmol/L, while less than 3.0 mmol/L [11, 22]. Differences in the baseline characteristics were analyzed by ANOVA test and post hoc least significant difference *t*-test, Kruskal–Wallis test and Nemenyi post hoc test, and the chi-squared test and partitions of the chi-squared test post hoc test. Multiple linear regression analysis was performed to identify independent factors for KBs. Binary logistic regression analysis was employed to determine whether different levels of KBs are independently associated with glomerulus, renal tubules, and renal arteries. The contingency coefficient C was calculated to quantify the relationship between KBs and glomerulus, renal tubules, and renal arteries. The sensitivity and specificity of the KB measurement at the diagnosis of renal function damage were calculated using the receiver operating characteristic (ROC) analyses. The best cut-off value was determined by the Youden index [sensitivity + specificity − 1]. *P* < 0.05 was considered statistically significant.

## 3. Results

### 3.1. Study Population Characteristics

The clinicopathological characteristics of 955 T2DM patients were categorized according to their levels of KBs as shown in Table 1. In the four groups, patients in the high-normal KB group demonstrated the highest eGFR (*P* < 0.01) and the lowest incidence rate of DKD (*P* < 0.05). Significant changes were observed in age, sex, smoking [23], drinking [24], heart rate (HR), fasting plasma glucose (FPG), 2-hour plasma glucose (2hPG), HbA1c, TG, albumin (ALB), *α*1-MG, and urine protein (PRO). However, no significant differences were observed in SBP, DBP, TC, LDL-C, BUN, SCr, UACR, TRU, and OADs. In addition, multiple linear regression analysis showed that sex, BMI, BUN, 2hPG, HR, and drinking were significantly associated with KBs in T2DM patients (all *P* < 0.05), and 2hPG was the most influence on KBs according to standardization coefficient (supplemental table 1).

### 3.2. Association of KB Concentration with DKD

**Figure 1:**
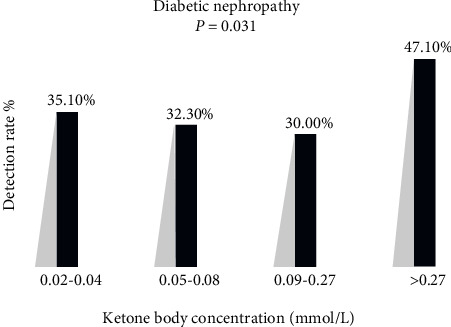
The incidence of diabetic kidney disease in different KB concentration-based groups: low-normal KB group, 35.10%; middle-normal KB group, 32.30%; high-normal KB group, 30.00%; and slightly elevated KB group, 47.10%; *P* = 0.031.

An association was found between the levels of KBs and DKD. A significant difference in the prevalence of DKD was observed in the study (low-normal KB group, 35.10%; middle-normal KB group, 32.30%; high-normal KB group, 30.00%; slightly elevated KB group, 47.10%; *P* = 0.031; Figure 1).

Table 2 shows the results of binary logistic regression analysis for DKD according to the concentration of KBs. In the unadjusted Model #1, diabetic patients in the normal KB groups (low-normal KB group: odds ratio (OR) = 0.683, *P* = 0.043; middle-normal KB group: OR = 0.631, *P* = 0.015; high-normal KB group: OR = 0.602, *P* = 0.004) demonstrated a significantly lower risk of DKD when compared to those patients in the slightly elevated KB group. After adjusting the age, duration of diabetes, SBP, DBP, HR, TC, TG, LDL-C, HbA1c, BUN, ALB, BMI, smoking, and drinking, Model #3 demonstrated that diabetic patients in the high-normal KB group had the lowest risk of DKD (OR = 0.435, *P* = 0.032).

### 3.3. Association of KB Concentration with Glomerulus, Renal Tubules, and Renal Arteries

**Figure 2:**
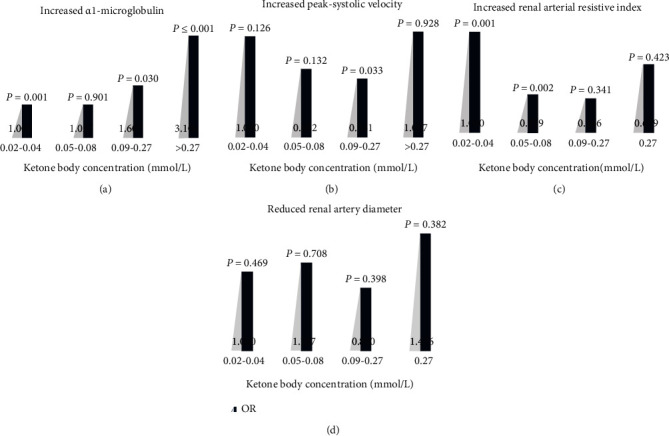
(a) Adjusted ORs of increased *α*1-microglobulin in different KB concentration-based groups. (b) Adjusted ORs of increased peak systolic velocity of renal artery in different KB concentration-based groups. (c) Adjusted ORs of increased renal arterial resistive index in different KB concentration-based groups. (d) Adjusted ORs of reduced renal artery diameter in different KB concentration-based groups. Adjusted for age, duration of diabetes, SBP, DBP, HR, TC, TG, LDL-C, HbA1c, BNU, ALB, BMI, smoking, and drinking.

In the present study, the OR of increased *α*1-MG was positively associated with KB concentration, and diabetic patients in the slightly elevated KB group have a significantly highest risk of increased *α*1-MG (OR = 3.106, *P* ≤ 0.001; Figure 2(a)) compared with the low-normal KB group after adjusting for the confounding factors. Renal vascular indicators were detected by color Doppler ultrasound in 455 T2DM patients. The binary logistic regression analyses indicated that T2DM patients in the high-normal KB group exhibited a significantly lower risk of increased PSV (OR = 0.581, *P* = 0.033; Figure 2(b)), and those in the middle-normal KB group had a remarkably lower risk of increased RI (OR = 0.379, *P* = 0.002; Figure 2(c)). There were no significant differences in other groups when compared with the low-normal KB group after adjusting for the abovementioned confounding factors. In addition, compared with the low-normal KB group, no significant relationship was observed between the KB concentration with the risk of reduced diameter of renal artery (middle-normal group: OR = 1.107, *P* = 0.708; high-normal group: OR = 0.800, *P* = 0.398; slightly elevated group: OR = 1.476, *P* = 0.382; Figure 2(d)). The relationship of KB levels was found with the prevalence of DKD (correlation coefficient = 0.096, *P* = 0.031), increased *α*1-microglobulin (correlation coefficient = 0.160, *P* ≤ 0.001), increased renal arterial resistive index (correlation coefficient = 0.212, *P* ≤ 0.001), and increased peak systolic velocity (correlation coefficient = 0.097, *P* = 0.245), showing the renal tubule is more influenced by KBs (Supplemental fig. 2). Furthermore, in the ROC curve of KBs for detection of renal function damage, the best KB cut-off value, as assessed by the Youden index, was 0.09 mmol/L, which provided a sensitivity of 36% and a specificity of 74% (Supplemental fig. 3).

## 4. Discussion

In the present study, DKD and PSV were found to be significantly decreased in the high-normal KB group; RI was notably reduced in the middle-normal KB group, *α*1-MG was positively associated with KB concentration, and the indicators of glomerulus, renal tubules, and renal arteries were all poor in the slightly elevated KB group, suggesting that there is no evidence indicating that slightly elevated circulating levels of KBs have a positive influence on patients with T2DM.

In the current research, individuals with slightly elevated circulating levels of KBs had the highest risk of DKD suggesting that a slight increase in KB level is a potential risk factor for DKD. Moreover, the eGFR value was the lowest in the slightly elevated KB group, suggesting that slightly elevated circulating levels of KBs showed no significant effect on renal blood flow. The results also unveiled that slightly elevated levels of KBs do not play a protective role in diabetic microangiopathy. This is inconsistent with the previous reported findings related to diabetic macroangiopathy, especially cardiovascular disease [25–28]. Brownlee has pointed out that excessive production of mitochondrial superoxide is a common mechanism in diabetes complications [29]. Additionally, diabetic macrovascular and microvascular complications often coexist in patients with T2DM, and the two often share common risk factors, such endothelial cell injury [30, 31]. However, it is highly essential to explore whether the behavior of endothelial cells in different areas is accompanied with a great heterogeneity [32]. This is an important mechanism for the slight elevation of KB levels accompanied with significant differences in microvascular and macrovascular complications. Other possible mechanism is that the high level of KBs is often associated with high blood sugar level, and the elevated levels of KBs activated varied harmful pathological complications, resulting in noxious stimuli that cause cell injury [8]. In addition, the result revealed that the prevalence of DKD and the value of eGFR shared the same trend of variability, and both showed a significant protective signal at high-normal levels of KBs and a negative effect at slightly elevated KB levels, while UACR and KB concentrations had no obvious relationship, suggesting that the KBs and eGFR are more strongly associated with the relationship between KBs and DKD. It is well known that RI, PSV, and eGFR were intimately linked with renal perfusion [33], and the study has shown a correlation between RI, PSV, and KBs, which also supports the above conclusions.

It is well known that the patients with DM are at high risk of developing atherosclerotic renal artery stenosis (RAS) [34]. However, there is lack of consensus on the relationship between the levels of KBs and renal artery. A number of scholars have demonstrated that slightly elevated circulating levels of KBs can reduce renal ischemia-reperfusion injury through anti-inflammatory effects, and it also plays a positive role in the treatment of acute kidney injury [5]. In the present study, it was revealed that heart rate showed association with KB concentration, while cases with high-normal levels of KBs had the lowest risk of increased PSV, and those with middle-normal levels of KBs had the lowest risk of increased RI [35]. Moreover, RI is not only a risk factor of glomerular and tubular damage and kidney disease progression but also a marker of endothelial dysfunction [36]. The results of the present study were not consistent with those reported previously, due to the following reasons. Firstly, diabetic heart is characterized by failure of insulin to increase glucose uptake and increasingly relies on free fatty acids (FFAs) as a source of energy, while KB as a supplementary source of energy plays a significant role in heart health [28, 37, 38]. Secondly, although the structure of the endothelium is similar in various vascular segments, the endothelial behavior in the endothelial territories is significantly different, which is independent of several factors [32]. Thirdly, hyperketonemia and hyperglycemia often coexist in T2DM patients, complicating the relationship between the levels of KBs and renal artery. Finally, the slightly elevated circulating levels of KBs resulted in macrophage and lymphocyte activation, increased risk of vascular inflammation, and perturbation of capillary endothelial cells [37, 38]. In addition, PSV and RI were associated with higher levels of Doppler ultrasound parameters of RAS; it is interesting that the distribution of PSV (lowest in high-normal range) and RI (lowest in middle-normal group) is different. The underlying reason for the inconsistency may be that RI reflects the macroscopic perfusion resistance of the distal vascular system of the monitoring point, while PSV reflects the hemodynamic parameters of the monitoring point and can be affected by the conditions of arteries upstream or downstream of a stenosis [35, 39]. Therefore, RI has a higher sensitivity to the effects of ketone bodies on renal arteries.

It is noteworthy that the renal tubule plays a pivotal role in the pathogenesis of DKD and actively participates in the progression of end-stage renal disease (ESRD) [1, 40]. Studies have demonstrated that podocyte damage and proximal tubule dysfunction biomarkers could be validated as a practical approach to the diagnosis of early DKD [32, 40]. Besides, *α*1-MG is important parameter for evaluating renal tubular injury, and its increased values reflect tubular damage and can be detected in early DKD [41]. SGLT-2 inhibitors are antidiabetic agents that reduce hyperglycemia by impairing glucose reabsorption in the proximal tubule of the kidney and increase glucosuria. SGLT2 inhibitors have shown to reduce glucotoxicity in isolated proximal tubule cells and also to attenuate the expression of markers of overall kidney damage in experimental animal models of diabetes [42–44]. In the current study, the value of *α*1-MG reached maximum when the KB level was slightly elevated, which indicated that in contrast to the complex multifactor renal protection mechanism of SGLT-2 inhibitors, a slightly elevated KB level does not play a role in kidney protection, while it is a significant contributor in increasing the risk of tubular injury.

In the present study, both the prevalence of DKD and the OR of renal artery Doppler ultrasound parameters have a U-shaped distribution with the KB concentration, whereas the OR of increased *α*1-MG was positively associated with KB concentration, with slightly elevated levels of KBs having the least benefit for the glomerulus, renal tubules, and renal arteries. The specific reasons for this distribution may be as follows. Firstly, DKD is classically regarded to be manifested as glomerulosclerosis, tubulointerstitial fibrosis, and renal vascular disease [45]. In addition, the different injury mechanisms of glomerulus, renal tubules, and renal arteries induce different degrees of damage and different features in various stages of kidney disease. Secondly, many studies have revealed that apart from serving as energy fuels, KBs play critical roles as modulators of inflammation and oxidative stress [4]. Within a certain range, as the concentration of KBs increases, its effect is enhanced, anything beyond that would lead to the protective effect decreased gradually. The optimal KB concentration of function protection varies among glomerulus, renal tubules, and renal arteries. This study demonstrated that when the KB concentration is lower than 0.09 mmol/L, it can be perceived as a cut-off for low risk of renal function damage. Thirdly, although the benefits of KBs have been confirmed, nevertheless excessive KBs can cause adverse effects. The elevated ketones in a manner that is detrimental and injurious inducing oxidative stress and eliciting inflammatory responses which interfere the normal cellular function and mediate cellular damage, also play a prominent role in the development of complications associated with diabetes [8]. In addition, the presence of elevated ketones with high glucose can engender more damage to the cells compared to that of just high glucose alone [8]. Finally, interestingly, some studies have clarified that there is a U-shaped relationship between HbA1c and mortality and when HbA1c levels between 7 and 8% seem to be associated with the highest survival rates in DKD patients [46]. The ACCORD, VADT, and ADVANCE studies, which primarily focused on intensive blood sugar control in patients with T2DM, did not find any benefits in clinical renal outcomes [47]. The conclusion is consistent with the results of KBs, suggesting that maintaining blood glucose and blood ketone levels to a certain extent has a more protective effect on DKD. We need more dynamic blood glucose indicators and more samples to explore the accurate ranges and specific mechanisms.

However, there is a limitation in the present study with regard to the lack of DKA patients' data and the pathological indexes. Besides, a number of patients missed undergoing vascular color Doppler ultrasound. Additionally, as this was a cross-sectional study, causal associations between the levels of KBs and kidney function in patients with T2DM could not be allowed. Hence, further in-depth studies should be conducted to confirm these findings and eliminate the abovementioned shortcomings.

The major strength of the present study was comprehensive assessment of the relationship between the levels of KBs and kidney function in patients with T2DM. We demonstrated that individuals with high-normal levels of KBs had the lowest risk of DKD and increased PSV; those with middle-normal levels of KBs had the lowest risk of increased RI, with a positive correlation between increased *α*1-microglobulin and KB concentration. In addition, the indicators of glomerulus, renal tubules, and renal arteries were all poor with slightly elevated circulating levels of KBs, and KB concentration lower than 0.09 mmol/L can be applied as the threshold for low risk of renal function damage. In summary, slightly elevated circulating levels of ketone bodies are not of benefit for kidney function in patients with type 2 diabetes mellitus. Therefore, the circulating levels of KBs can be used for detecting and predicting the kidney function in patients with T2DM.

## Figures and Tables

**Table 1:** The 955 T2DM patients' clinicopathological characteristics stratified by serum ketone body concentration.

Ketone body concentration (mmol/L)	0.02-0.04	0.05-0.08	0.09-0.27	>0.27	*P*
No of subjects	350	260	260	85	
Age (years old)	63.64 ± 11.26	61.05 ± 12.04^†^	60.12 ± 14.57^†^	61.29 ± 14.88	0.025^∗^
Male (*n* (%))	163 (46.6%)	143 (55.0%)	165 (63.5%)	58 (68.2%)	≤0.001^∗^
Duration of diabetes (year)	10.50 ± 7.72	9.90 ± 7.21	10.06 ± 7.66	8.89 ± 6.91	0.339
Smoker	57 (16.3%)	47 (18.1%)^†^	69 (26.5%)^‡^	21 (25.0%)^†§^	0.009^∗^
Alcohol	14 (4.0%)	16 (6.2%)^†^	20 (7.8%)^†^	11 (13.1%)^‡§^	0.015^∗^
BMI (kg/m^2^)	24.71 ± 3.83	25.05 ± 4.54	24.58 ± 3.94	23.36 ± 3.95^†‡§^	0.023^∗^
Prevalence of hypertension *n* (%)	211 (60.5%)	146 (56.2%)	142 (54.6%)	47 (56.0%)	0.495
SBP (mmHg)	134.9 ± 22.2	134.8 ± 19.8	135.8 ± 18.8	135.3 ± 22.6	0.941
DBP (mmHg)	77.9 ± 11.4	78.15 ± 10.6	79.51 ± 11.1	79.58 ± 10.7	0.253
HR (bpm)	71.10 ± 12.31	72.08 ± 11.17	74.45 ± 12.76^†‡^	77.57 ± 13.65^†‡^	≤0.001^∗^
FPG (mmol/L)	7.26 ± 3.26	7.99 ± 3.14^†^	8.73 ± 3.83^†‡^	8.79 ± 4.37^†^	≤0.001^∗^
2hPG (mmol/L)	12.05 ± 4.19	12.25 ± 4.11	13.61 ± 4.13^†‡^	13.37 ± 5.14^†^	≤0.001^∗^
HbA1c (%)	8.55 ± 2.27	9.04 ± 2.40^†^	9.57 ± 2.61^†‡^	9.65 ± 2.77^†^	≤0.001^∗^
TG (mmol/L)	1.33 (0.90-1.97)	1.36 (0.97-2.07)	1.40 (1.02-2.18)	1.16 (0.84-1.87)	0.032^∗^
TC (mmol/L)	4.34 ± 0.98	4.42 ± 1.23	4.57 ± 1.39	4.44 ± 1.65	0.159
LDL (mmol/L)	2.67 ± 0.84	2.80 ± 1.05	2.85 ± 1.07	2.71 ± 1.05	0.125
ALB (g/L)	39.04 ± 4.18	39.99 ± 4.52^†^	39.59 ± 4.86	37.46 ± 6.06^†‡§^	0.001^∗^
BUN (mmol/L)	6.42 ± 3.86	6.44 ± 3.52	6.23 ± 3.19	6.64 ± 4.82	0.808
SCr (umol/L)	60.85 (49.38-76.70)	59.80 (47.93-73.95)	61.70 (49.08-74.00)	65.20 (46.30-83.00)	0.572
eGFR (mL/min)	92.21 ± 26.56	96.09 ± 26.52	98.33 ± 28.00^†^	92.89 ± 31.36	0.009^∗^
UACR (mg/g)	13.65 (6.27-94.39)	13.49 (6.45-62.62)	16.81 (7.42-83.76)	28.80 (10.98-145.92)	0.055
*α*1-MG (mg/L)	10.20 (4.19-20.50)	9.18 (4.30-21.05)	11.10 (4.78-24.05)	21.10 (8.93-55.47)^†‡§^	≤0.001^∗^
U-IgG (mg/L)	8.42 (4.41-23.98)	6.82 (4.48-15.92)	7.31 (4.27-19.70)	14.20 (5.36-45.92)^‡§^	0.020^∗^
TRU (mg/L)	9.97 (4.31-36.67)	6.77 (3.34-22.45)	8.96 (4.00-46.20)	10.07 (3.43-61.22)^†‡^	0.386
DKD (%)	123 (35.1%)	84 (32.3%)	78 (30.0%)	40 (47.1%)^†‡§^	0.031^∗^
Increased*α*1-MG	110 (36.3%)	79 (32.8%)^†^	94 (41.2%)^‡^	45 (62.5%)^†‡§^	≤0.001^∗^

PRO					0.006^∗^
-	223 (67.0%)	165 (65.5%)	160 (66.4%)	38 (46.9%)	
+	110 (33.0%)	87 (34.5%)	81 (33.6%)	43 (53.1%)^†‡§^	

Reduced renal artery diameter (%)	122 (70.5%)	83 (69.7%)	81 (63.8%)	27 (75.0%)	0.489
Increased PSV (%)	81 (48.5%)	46 (40.4%)	48 (38.7%)	17 (51.2%)	0.245
Increased RI (%)	104 (62.3%)	43 (37.7%)	75 (60.5%)^†‡^	14 (42.4%)^†‡^	≤0.001^∗^

Type of antidiabetic therapy					0.236
None	38 (10.9%)	25 (9.6%)	33 (12.7%)	16 (19.0%)	
OADs	165 (47.4%)	144 (55.4%)	133 (51.2%)	35 (41.7%)	
Insulin	33 (9.5%)	26 (10.0%)	23 (8.8%)	9 (10.7%)	
OADs + insulin	112 (32.2%)	65 (25.0%)	71 (27.3%)	24 (28.6%)	

Post hoc analysis: ^†^compared with the ketone body concentration 0.02-0.04 group, *P* < 0.05; ^‡^compared with the ketone body concentration 0.05-0.08 group, *P* < 0.05; ^§^compared with the ketone body concentration 0.09-0.27 group, *P* < 0.05. SBP: systolic blood pressure; DBP: diastolic blood pressure; HR: heart rate; FPG: fasting plasma glucose; 2hPG: 2 h 75 g oral glucose tolerance test plasma glucose; HbA1c: glycated hemoglobin; TG: triglycerides; TC: total cholesterol; LDL-C: low-density lipoprotein cholesterol; BMI: body mass index; SCr: serum creatinine; GFR: glomerular filtration rate; UACR: urinary albumin-to-creatinine ratio; *α*1-MG: *α*1-microglobulin; U-IgG: urinary immunoglobulin G; TRU: urinary transferrin; PRO: urine protein; DKD: diabetic kidney disease; PSV: peak systolic velocity; RI: resistive index; OADs: oral antidiabetic drugs. ^∗^*P* < 0.05.

**Table 2:** The binary logistic regression analysis of DKD according to KB concentration.

Ketone body concentration (mmol/L)	Model 1		Model 2		Model 3	
	*P*	OR	*P*	OR	*P*	OR
0.02-0.04	0.043^∗^	0.683	0.046^∗^	0.470	0.057	0.484
0.05-0.08	0.015^∗^	0.631	0.064	0.491	0.070	0.495
0.09-0.27	0.004^∗^	0.602	0.025^∗^	0.424	0.032^∗^	0.435
>0.27	0.034^∗^	Ref	0.160	Ref	0.191	Ref

Model 1: unadjusted; Model 2: adjustment for sex, age, duration, SBP, DBP, HR, TC, TG, LDL, HbA1c, BUN, ALB, and BMI; Model 3: Model 2 + additional adjustment for consumption of tobacco and alcohol. ^∗^*P* < 0.05.

## Data Availability

The data used and/for analyzed during the current study are available from the corresponding author on reasonable request.

## Supplementary Materials

Supplementary Materials Supplemental Table 1: assessment of affected factors in KBs by multiple linear regression. Supplemental Figure 1: flowchart of patient selection. Supplemental Figure 2: the contingency coefficient (*C*) is used to evaluate the association of KB concentration with glomerulus, renal tubules, and renal arteries. Supplemental Figure 3: ROC analysis of KBs to indicate renal function damage for patients with type 2 diabetes mellitus.
