# Supplementary material for: The Value of Ketone Bodies in the Evaluation of Kidney Function in Patients with Type 2 Diabetes Mellitus
Source: J Diabetes Res. 2021 Apr 10;2021:5596125. doi: 10.1155/2021/5596125 (PMC8055418; doi:10.1155/2021/5596125)
Supplement: Supplementary Materials — Supplemental Table 1: assessment of affected factors in KBs by multiple linear regression. Supplemental Figure 1: flowchart of patient selection. Supplemental Figure 2: the contingency coefficient (C) is used to evaluate the association of KB concentration with glomerulus, renal tubules, and renal arteries. Supplemental Figure 3: ROC analysis of KBs to indicate renal function damage for patients with type 2 diabetes mellitus. [file 5596125.f1.zip › Supplemental fig. 3.docx]

**Supplemental fig. 3** ROC analysis of KBs to indicate renal function damage for patients with type 2 diabetes mellitus.
