# Supplementary material for: The Value of Ketone Bodies in the Evaluation of Kidney Function in Patients with Type 2 Diabetes Mellitus
Source: J Diabetes Res. 2021 Apr 10;2021:5596125. doi: 10.1155/2021/5596125 (PMC8055418; doi:10.1155/2021/5596125)
Supplement: Supplementary Materials — Supplemental Table 1: assessment of affected factors in KBs by multiple linear regression. Supplemental Figure 1: flowchart of patient selection. Supplemental Figure 2: the contingency coefficient (C) is used to evaluate the association of KB concentration with glomerulus, renal tubules, and renal arteries. Supplemental Figure 3: ROC analysis of KBs to indicate renal function damage for patients with type 2 diabetes mellitus. [file 5596125.f1.zip › Supplemental table 1.docx]

**Supplemental table. 1** Assessment of affected factors in KBs by multiple linear regression

|  | P values | **β** (95%CI) | Standardized coefficient |
| --- | --- | --- | --- |
| Age | 0.075 | -0.002(-0.004-0.000) | -0.131 |
| Duration of diabetes | 0.705 | 0.000(-0.003-0.002) | -0.019 |
| Sex (female vs. male) | 0.007* | -0.051(-0.089-(-0.014)) | -0.152 |
| BMI | 0.042* | -0.005(-0.009-0.000) | -0.099 |
| Smoker | 0.299 | -0.022(-0.064-0.020) | -0.055 |
| Alcohol | 0.011* | 0.078(0.018-0.138) | 0.126 |
| TG | 0.383 | 0.004 (-0.005-0.012) | 0.046 |
| TC | 0.262 | 0.009(-0.006-0.023) | 0.064 |
| FPG | 0.064 | 0.005(0.000-0.010) | 0.112 |
| 2hPG | ≤0.001* | 0.007(0.004-0.011) | 0.192 |
| HbA1c | 0.716 | -0.002(-0.010-0.007) | -0.023 |
| SBP | 0.484 | 0.000(-0.001-0.001) | 0.039 |
| DBP | 0.964 | 0.000(-0.002-0.002) | -0.002 |
| HR | 0.002* | 0.002(0.001-0.003) | 0.148 |
| ALB | 0.236 | -0.003(-0.007-0.002) | -0.069 |
| BUN | 0.017* | -0.011(-0.020-(-0.002)) | -0.181 |
| SCr | 0.075 | 0.001(0.000-0.001) | 0.166 |
| eGFR | 0.187 | -0.001(-0.002-0.000) | -0.127 |
| UACR | 0.181 | 0.000(-0.001-0.001) | -0.087 |

The correlation between KBs and variables was evaluated by standardized coefficient. BMI, body mass index; TG, triglycerides; TC, total cholesterol; FPG, fasting plasma glucose; 2hPG, 2-h 75-g oral glucose tolerance test plasma glucose; HbA1c, glycated hemoglobin; SBP, systolic blood pressure; DBP, diastolic blood pressure; HR, heart rate; BUN, blood urea nitrogen; SCr, serum creatinine; GFR, glomerular filtration rate; UACR, urinary albumin-to-creatinine ratio.

* P < 0.05.
